# Imaging and spectroscopic methods to investigate adult neurogenesis *in vivo*: New models and new avenues

**DOI:** 10.3389/fnins.2022.933947

**Published:** 2022-08-05

**Authors:** Nathalie Just, Pierre-Marie Chevillard, Martine Migaud

**Affiliations:** ^1^Danish Research Centre for Magnetic Resonance, Center for Functional and Diagnostic Imaging and Research, Copenhagen University Hospital Amager og Hvidovre, Hvidovre, Denmark; ^2^Physiologie de la Reproduction et des Comportements, Centre INRAE Val de Loire, CNRS, IFCE, INRAE, and Université de Tours, Nouzilly, France

**Keywords:** adult neurogenesis, MRI, animal models, human brain, controversy, diffusion, MRS

## Abstract

Adult neurogenesis (AN) can be defined as the birth and development of new neurons in adulthood. Until the 1990s, AN was deemed not to happen after birth. Gradually, several groups demonstrated that specific zones of the brain of various species had a neurogenic potential. AN could be the key to treating a large range of neurodegenerative, neuropsychiatric, and metabolic diseases, with a better understanding of the mechanisms allowing for regeneration of new neurons. Despite this promising prospect, the existence of AN has not been validated *in vivo* in humans and therefore remains controversial. Moreover, the weight of AN-induced plasticity against other mechanisms of brain plasticity is not known, adding to the controversy. In this review, we would like to show that recent technical advances in brain MR imaging methods combined with improved models can resolve the debate.

## Introduction

During the twentieth century, the prevalent dogma was that there was no generation of new neurons after birth. Then, gradually, different studies showed that adult neurogenesis (AN) induced plasticity in some specific regions of the brain of various species including humans. Changes occurred following learning, exercise, environmental enrichment, or stress. If validated, AN could have enormous implications for human health (Gage, 2019).

Despite more than 3 decades of research, the impact of AN on human health remains controversial because the *in-vivo* functional and metabolic roles of AN have not been established prompting to the non-existence of AN in the brain of adult humans (Sorrells et al., 2018). In their review of the current status of research on AN based on two opposite views on the existence of AN in the human brain, Kempermann et al. (2018) clearly pointed at “a clear need for additional ways to study the generation of new neurons in adult humans” and concluded against “the categorical claim that there is no AN in the human hippocampus” owing to the development of more quantitative analyses and *in vivo* methods. Here, we argue that there exists an arsenal of quantitative neuroimaging tools, which could be used efficiently to validate the presence or not of AN in the brain of adult humans. The aim of this mini review is not to review once more the history of AN, as this has been conducted on many occasions (Kuhn et al., 2018; Toda and Gage, 2018; Fares et al., 2019). In this review, we propose to discuss the wealth of existing quantitative magnetic resonance methodologies, which could be very efficient in detecting AN *in vivo*. Moreover, we suggest that the combination of these structural, functional, and metabolic quantitative MR techniques and their translational nature may help the validating procedure of AN in the human brain.

## A definition of adult neurogenesis

AN is the process by which new neurons are generated from neuronal stem cells in adults. This process involves the development of neural stem cells into progenitor cells their proliferation, differentiation, migration, and integration into existing neurons, and finally their maturation. It happens essentially during some activities such as exercise. Three major neurogenic niches have been identified in various species: the subgranular zone (SGZ), the subventricular zone (SVZ) of the hippocampus, and the olfactory bulb (Jurkowski et al., 2020). In addition, other areas of the brain have shown some neurogenic potential such as the striatum (Ernst et al., 2014) and the hypothalamus (Migaud et al., 2015).

## The existence of AN in the human brain remains controversial

AN has mainly been evaluated and validated using invasive and/or post-mortem methodologies. Among them, traditional immunohistochemical methods have been used in many animal models such as fish, frogs, birds, hamsters, rodents, monkeys, and sheep and the post-mortem human brain. Notably, bromodeoxyuredine (BrdU) was extensively used in the late 1990s to demonstrate the presence of AN in post-mortem SVZ brain samples of patients with cancer (Eriksson et al., 1998).

The ability of invasive methodologies to detect and quantify the presence of AN depends on the stage of the neurogenic process as well as the species on which they were tested. Numerous studies have been performed in the rodent brain, which is unfortunately not a representative model owing to important genetic differences and variations in cell type, cell growth, brain size, and development time to adulthood. The time to adulthood constitutes one of the main arguments against the existence of AN in the human brain, with studies showing a significant drop in the generation of new neurons in the dentate gyrus of the hippocampus of humans during the first years of life. Thus the conclusion to that AN at adulthood in humans does not exist (Sorrells et al., 2018). Despite these results, earlier ^14^C dating had demonstrated the existence of AN into the fifth decade of life in the human hippocampus (Spalding et al., 2013) but not in the olfactory bulb (Bergmann et al., 2012). Interestingly, these findings hinted at other mechanisms of brain plasticity in large brains such as different cytoarchitectural organizations of neurogenic niches and different migration paths compared to rodents. Notably, the capacity of astrocytes to act as neuronal stem cells (NSCs) was shown. It can therefore be envisaged that specific cell populations might be dependent on specific types of brain activities and mechanisms or disruptions of brain activities necessitating different sensitivities of detection techniques as well as new biomarkers.

## AN has been mainly investigated with immunohistochemical methods and in post-mortem samples

The development of newborn neurons from NSCs until they integrate either the hippocampal or the hypothalamic circuitry passes through multiple development stages, each of which depends on the expression of specific protein markers described in-depth in many articles (Kuhn et al., 2016). AN has therefore a time-dependent and functional development, which is difficult to observe longitudinally by immunohistochemical studies. Thus, glial fibrillary acidic proteins (GFAPs), Nestin, Sox2, and bromodeoxyuridine (BrdU), which has been widely used to detect the presence of neurogenesis and represents the gold standard for validating the existence of AN, are often used to recognize proliferation stages. Doublecortin (DCX) is used as a marker of differentiation and/or migration. Finally, calretinin, Neun, and calbindin expressions can be useful for detection of new neurons and their integration to local circuitry (Kozareva et al., 2019).

Despite their repeated usage to demonstrate proliferation, differentiation, or migration of cells in AN, many immunohistochemical markers present confounding effects. For example, BrdU labeling performance depends on the status of the blood brain barrier or can label phenomena other than proliferation (Sierra et al., 2011). The specificity of DCX as a marker of AN has also been challenged (Sierra et al., 2011), while the expression of other antibodies and markers of proliferation must be performed with care since non-neurogenic cells may also be labeled.

## An existing panel of *in vivo* MRI techniques

To establish the existence of AN *in vivo* and demonstrate its impact on human health, it is necessary to perform non-invasive *in vivo* neuroimaging. Magnetic resonance imaging (MRI) is, by far, one of the most translatable imaging techniques currently available for estimation of non-invasive biomarkers of development and progression of diseases and for investigation of responses to treatment in various species. MRI offers a large panel of qualitative, semi-quantitative, and quantitative parameters for visualization and understanding of both healthy and pathological features.

For the past 20 years, crucial technological developments of MRI machines have permitted important advances of structural, functional, and metabolic imaging methods that are now widely used in both experimental and clinical settings. As a matter of fact, a wide range of identical imaging sequences [for dynamic contrast-enhanced (DCE), dynamic susceptibility contrast (DSC) MRI, for diffusion tensor (DTI) MRI, for blood oxygen level-dependent functional MRI (BOLD fMRI), for perfusion MRI etc.] exists for small animals and for humans, while more and more animal-dedicated scanners propose similar hardware and software functionalities. Moreover, subsequent image analysis of animal or human scans often uses identical platforms, although programs need to be adapted.

Of course, direct detection of AN *in vivo* necessitates acquisition of images at cellular resolution. With conventional magnetic resonance imaging (MRI) techniques, the *in vivo* in plane spatial resolution is rarely below 100 μm^2^. Direct detection of AN is therefore difficult with MR techniques, and many studies mention possible effects of AN without demonstrating its presence. In Table 1, we enumerated studies using MR techniques to evaluate the presence in or effects of AN on both animals and humans. This summary is not exhaustive but demonstrates the lack of direct *in vivo* MR studies on AN.

**Table 1:** MR publications on adult neurogenesis.

**MR technique**	**Publications**	**Species**
Reviews	Ho et al. (2013). *In vivo* imaging of adult human hippocampal neurogenesis: progress, pitfalls and promise.	Mainly humans
	Couillard-Despres and Aigner (2011). *In vivo* imaging of adult neurogenesis.	Rodents
	Couillard-Despres et al. (2011). *In vivo* monitoring of adult neurogenesis in health and disease.	Rodents
	Lucassen et al. (2020). Adult neurogenesis, human after all (again): Classic, optimized, and future approaches.	
Structural MRI/VBM	Franklin et al. (2013). A VBM study demonstrating 'apparent' effects of a single dose of medication on T1-weighted MRIs.	Humans
	Killgore et al. (2013). Physical exercise habits correlate with gray matter volume of the hippocampus in healthy adult humans.	Humans
	Pajkert et al. (2020). Early volumetric changes of hippocampus and medial prefrontal cortex following medial temporal lobe resection.	Humans
	Sasaki et al. (2015). Specific regions display altered gray matter volume in μ-opioid receptor knockout mice: MRI voxel-based morphometry.	Mice
BOLD fMRI/	Gao et al. (2021). Blockade of Indoleamine 2, 3-dioxygenase 1 ameliorates hippocampal neurogenesis and BOLD-fMRI signals in chronic stress precipitated depression.	Rodents
Resting-State fMRI	van der Marel et al. (2015). Effects of long-term methylphenidate treatment in adolescent and adult rats on hippocampal shape, functional connectivity and adult neurogenesis.	Rodents
	Chevillard et al. (2021). Hypercapnic challenge, BOLD fMRI and immunohistochemistry to examine the *in-vivo* presence of adult neurogenesis in the sheep hypothalamus.	Sheep
CBV-CBF	Pereira et al. (2007). An *in vivo* correlate of exercise-induced neurogenesis in the adult dentate gyrus.	Humans and rodents
	Taupin (2009). Magnetic resonance imaging for monitoring neurogenesis in the adult hippocampus.	Humans and rodents
Diffusion MRI	Sierra et al. (2015). Imaging microstructural damage and plasticity in the hippocampus during epileptogenesis.	Rodents
	Pérès et al. (2018). Longitudinal Study of Irradiation-Induced Brain Microstructural Alterations With S-Index, a Diffusion MRI Biomarker, and MR Spectroscopy.	Rodents
	Treit et al. (2018). High resolution *in-vivo* diffusion imaging of the human hippocampus.	Humans
	Rutland et al. (2019). Hippocampal subfield-specific connectivity findings in major depressive disorder: A 7 Tesla diffusion MRI study.	
	Bálentová et al. (2017). Effect of whole-brain irradiation on the specific brain regions in a rat model: Metabolic and histopathological changes.	Rodents
MRS	Zhang et al. (2017). NSCs promote hippocampal neurogenesis, metabolic changes and synaptogenesis in APP/PS1 transgenic mice.	Rodents
	Just et al. (2021a). Photoperiodic Regulation of hypothalamic metabolism: a preliminary single voxel Magnetic Resonance Spectroscopy investigation at 3T.	Sheep
	Manganas et al. (2007). Magnetic resonance spectroscopy identifies neural progenitor cells in the live human brain.	Rodents and Humans

A wide range of MR techniques has been developed for the past 30 years, allowing for characterization and relative or absolute quantification of biomarkers or surrogate markers of various structural, functional, or even metabolic changes occurring in the brain *in vivo*. Among them, vascular markers such as cerebral blood flow (CBF) and cerebral blood volume (CBV) have been used as potential surrogate markers of AN both in the rodent and human hippocampi after prolonged exercise (Pereira et al., 2007). Thus, despite the lack of high-resolution images, the impact of AN on brain physiology is measurable and quantifiable in a translational fashion by MRI. Moreover, MR measurements can be repeated longitudinally on same subjects across years. Many other MR techniques can be used to probe the presence of AN [blood oxygen level-dependent (BOLD) functional MRI, diffusion MRI, or MR spectroscopy (MRS)] but have been scarcely used in this context (Table 1). Unfortunately, to date, most studies remain correlative, where MR parameters are correlated to BrdU or DCX labeling to prove the existence and impact of AN. Disentangling the effects of AN on MR parameters from other effects (i.e., exercise and disease) remains challenging. In addition, AN may consist of generation of only a few cells to which MR parameter changes may not be sensitive.

## Animal models

Rodent models are widely used in MRI owing to their wide availability, including transgenic models, as well as their flexibility, homogeneity, ease of use for preclinical imaging studies and the important knowledge accumulated over the years on them. They provide an important source of valuable knowledge on human disease allowing cross-correlations of MRI with cellular and molecular parameters and thus bring insights into the underlying mechanisms of various disorders. Nevertheless, their physiology, their behavior, and their development from birth to adulthood within a few months as opposed to several years in the human, do not always make valid models of them. The results obtained on their lissencephalic brain are difficult to transpose to humans and, the lack of specific features or symptoms, especially in mental disorders (in schizophrenia, autism) make them irrelevant for pharmaco-therapeutic challenges. In terms of MRI, the differences in spatial and temporal resolutions can be difficult to transpose to the humans in the clinical setting despite the use of higher magnetic field strengths for research only purposes. Finally, physiological and behavioral responses to anesthesia in rodents may differ substantially from the humans, which nevertheless are rarely anesthetized for routine MRI scanning.

On the other hand, non-human primates (NHPs) are considered the most relevant animal model for translational purposes, being the closest animal models to humans regarding their genetic background, physiology, and behavior. Most often, they are considered as the last criterion for establishing the efficacy of a drug before transition to humans. However, recent studies have also provided evidence that NHP models have also provided disappointing contributions to medical advancements. Moreover, ethical issues such as suffering and deaths of NHPs in biomedical research are frequently raised. The validity of some studies regarding cognition and behavior assessments of captive NHPs has also been questioned.

Despite the ethical issues mentioned previously, establishment of relevant NHP models of various diseases in combination with non-invasive MRI techniques is a growing area of research holding great promises for development and assessment of various therapeutic protocols. Moreover, MRI investigation of NHPs can be useful to validate the characterization of deeper brain structures and specific nuclei (Zitella et al., 2015) including in routinely used clinical scanners at a field strength of 3T and using whole-body MR features such as parallel imaging further enforcing the usefulness of NHPs for translational research (Li and Zhang, 2017). In addition, NHP models can be used effectively to investigate neural circuitry with cutting-edge technologies such as optogenetics (Senova et al., 2018), which that can be coupled with MRI techniques to identify specific anatomical targets for further stimulation therapy.

In between rodent and NHP animal models, there exists a wide range of other animal models that already showed an interesting potential for translational biomedical research. This is the case of the ovine model, which can be an interesting alternative to NHPs for MRI techniques (Ella et al., 2017; Just et al., 2021a).

**Figure 1:**
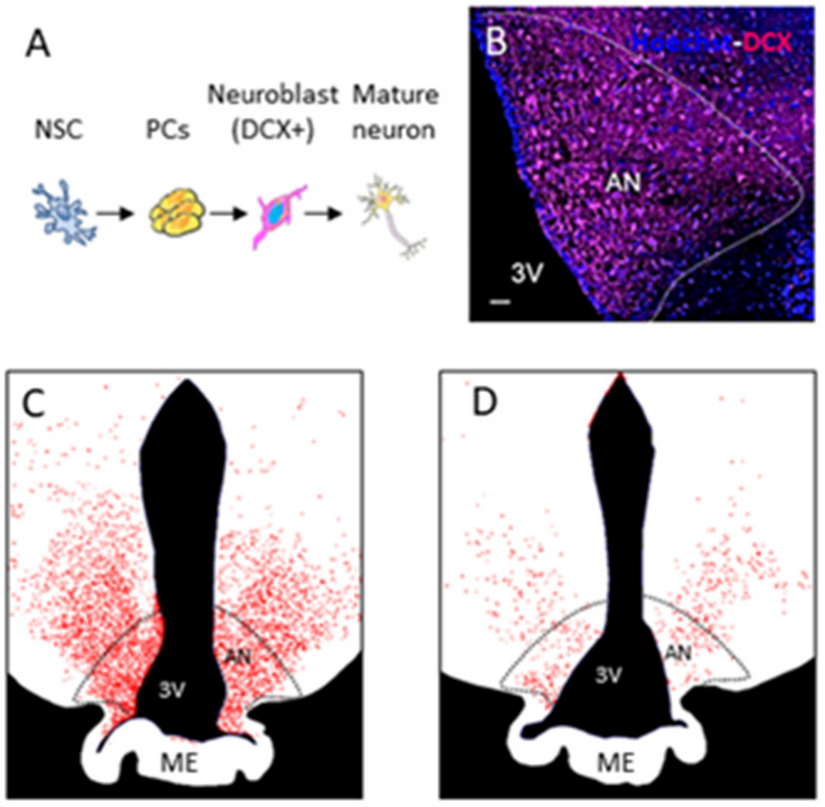
Adult neurogenesis in the hypothalamus of sheep. **(A)** Different stages of adult neurogenesis from neural stem cells (NSCs) to DX-positive mature neurons. **(B)** Photomicrograph of DCX-positive staining in the arcuate nucleus of the sheep hypothalamus. **(C,D)** Drawings showing variations in DCX + staining during photoperiod.

Sheep have reproducibly demonstrated AN in the hypothalamus (Migaud et al., 2015; Butruille et al., 2018). Compared to other species with a gyrencephalic brain, sheep are sensitive to photoperiod. The photoperiod represents the ratio of day to night length, which regulates the reproduction cycle of this species (Malpaux et al., 2001). BrdU and DCX staining of the median eminence and arcuate nucleus of the hypothalamus in sheep showed that AN occurs during the shortest days of the year (from the end of September to the start of January) corresponding as well to the period of reproduction. Figure 1B shows a photomicrograph example of DCX-positive staining in the arcuate nucleus of the sheep hypothalamus followed by drawings showing variations in DCX+ staining during photoperiod (Figures 1C,D) (Migaud et al., 2015; Chevillard et al., 2022). In these animals, AN can be seen as a naturally occurring phenomenon, which confers an increased interest to this animal model in addition to their gyrencephalic brain, extended time to adulthood (3–4 years after birth), and possibility to be examined under similar conditions with identical imaging sequences to humans in clinical MR scanners (Just et al., 2021a).

## Multi-MR techniques for *in vivo* detection of AN in the sheep brain

AN is a dynamic process involving multiple structural, vascular, functional, and metabolic changes across time. Interestingly, several MR techniques are designed to probe these changes. Unfortunately, only hypotheses on their potential role on the detection of AN have been emitted.

## Voxel-based morphometry (VBM) and adult neurogenesis

**Figure 2:**
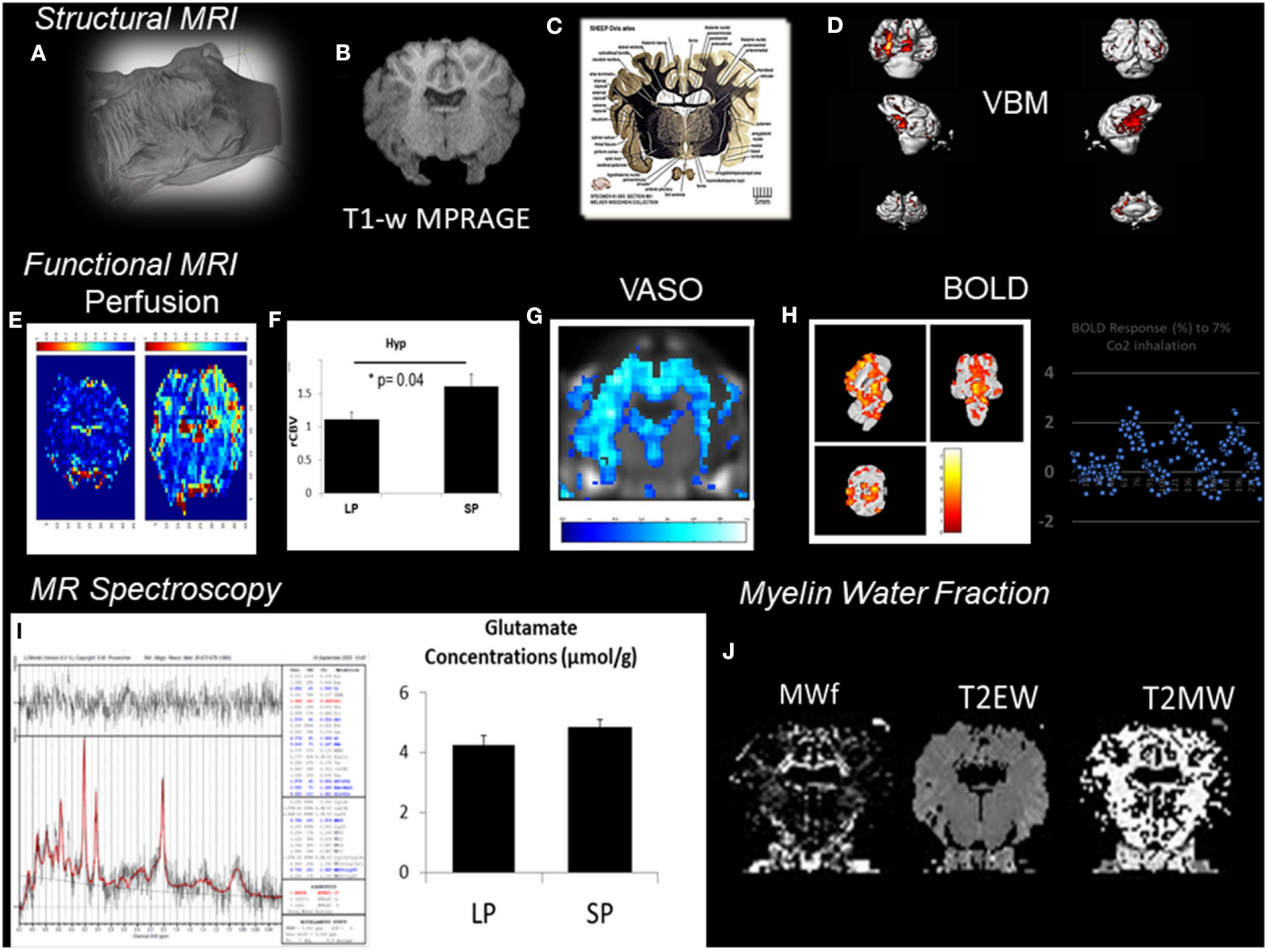
Patchwork of different MR techniques used to explore the response of different structural and functional MR parameters to AN in the sheep hypothalamus during photopriodism. **(A)** Structural MR imaging and sensitivity to AN in the sheep hypothalamus. **(B)** VBM analysis conducted from the segmentation of MPRAGE images (0.5 × 0.5 × 0.2 mm^3^). **(C)** Labeled fiber staining from the Sheep Brain Atlas (Michigan State University) showing the hypothalamus of the sheep with specific nuclei such as the arcuate nucleus. **(D)** VBM analysis detected significant structural changes occurring during the short days of the year (SP, short period) during AN compared to the long days of the year (LP, long period). **(E)** Functional MR imaging and sensitivity to AN rCBVmaps. **(F)** Comparison between LP and SP in the hypothalamus show a significant increase during SP (see Just et al., 2021b for methods). **(G)** Example of vascular occupancy (VASO) map during C02 inhalation. **(H)** BOLD fMRI upon hypercapnic challenge during SP (Chevillard et al., 2021). **(I)** ^1^H-MRS of the hypothalamus demonstrated activation in hypothalamic areas and corresponded well to changes in glutamate. **(J)** Example of Myelin water fraction (MWf) and T2 maps. These examples were extracted from data, which are still under analysis but appear to show some consistent response among all the different MR modalities used with structural, functional, and hemodynamic changes occurring during the period of AN.

In VBM, high-resolution structural volumes of the brain are compared statistically to draw conclusions on differences in brain regions between groups. Preliminary examples of a VBM analysis (Figure 2) conducted from segmentation of MPRAGE images of the sheep brain (Figure 2B) show significant structural changes occurring during the short days of the year (SP, short period) during AN compared to the long days of the year (LP, long period). We expected changes to arise from the arcuate nucleus and the median eminence of the hypothalamus (Figure 2C; The Sheep Brain Atlas, Michigan State University) due to ongoing AN. Several studies demonstrated that VBM could be advantageous for the analysis of the human brain in health and disease but the nature of the cellular changes underlying the signal of structural images has often been speculated to come from AN, endothelial cell or glial cell proliferation as well as changes in dendritic spine density but rarely probed for these effects. For example, Killgore et al. (2013) correlated total minutes of weekly exercise to changes in hippocampal volume evaluated with VBM. They associated their findings to AN. Franklin et al. (2013), on the other hand, showed that vascular effects could easily confound VBM findings in the hippocampus demonstrating that changes in neural mechanisms should always be tested to confirm that they could be the source of structural changes.

## Blood oxygen level-dependent fMRI, perfusion MRI, and other functional MR techniques

The BOLD signal represents one of the acknowledged proxies for neuronal activity. As such, it could be thought of as one of the best ways to investigate AN. Again, the potential of the BOLD signal as a surrogate marker of AN has been speculated on many instances with very few attempts to probe this hypothesis. Interestingly if one types “Neurogenesis and BOLD-fMRI” on PubMed, almost only rodent studies are found, although human studies exist and have compared BOLD functional responses in the hippocampus in elderly populations vs. younger populations showing CA3/dentate functional deficits related to memory impairments in healthy elderly subjects (Yassa and Stark, 2011). Although authors compared their findings to animal studies also using a pattern separation task in which the role of newborn granule cells was demonstrated (Clelland et al., 2009), the potential role of AN was not reported.

In contrast to VBM, the BOLD signal has been extensively examined for many years, with many studies linking cellular changes to BOLD responses (Kida and Hyder, 2006; Just et al., 2013). Nevertheless, the BOLD signal corresponds to a hemodynamic response, which includes changes in cerebral blood volume (CBV), cerebral blood flow (CBF), and rate of oxygen consumption (CMRO_2_) and describes neuronal activity in an indirect manner by neurovascular coupling. The underpinnings of BOLD responses remain difficult to apprehend with conventional methods. Therefore dissociating the BOLD signals due to newborn neurons from already existing neurons requires cutting-edge techniques involving the use of ultra-high magnetic fields as well as the combination of MR techniques with genetic tools (i.e., optogenetics or calcium imaging) to access the single cell function (Chen et al., 2019), which obviously cannot be performed *in vivo* in the human brain. Parallel neuroimaging studies can, however, be conducted on animal models and humans as was performed with MRI CBV (Pereira et al., 2007). CBV measurements were performed longitudinally in mice exercising daily demonstrating significant increases in the dentate gyrus (DG), which correlated with BrdU expression and validated the presence of AN. In a cohort of adult human subjects, CBV in the DG followed the same trend while CBF was also increased and correlated with cognition, suggesting a potential role of AN by extrapolation of animal results. Recently, Steventon et al. (2020) examined whether CBF augmentation in the hippocampus following long-term exercises had a mechanistic vascular origin using both ASL and cerebrovascular reactivity (CVR) to CO_2_ inhalation. They showed that CBF changes due to exercise were more likely to arise from adaptive metabolic changes, which could trigger AN and vascular plasticity, although these were only demonstrated through similar studies. Alternatively, CBV is correlated to angiogenesis, which is coupled to progenitor proliferation (Chevillard et al., 2022). Steventon et al. (2020) suggested that vascular plasticity changes could arise from mature neurons since vascular endothelial growth factor changes occur prior to new neurons becoming functional. However, perfusion changes due to exercise that were measured without concurrent changes in CBF and BOLD signal could also be due to the earlier proliferation stages of AN, whereas BOLD signal and CBF changes would occur in a later stage when a neural network is established (Ho et al., 2013). Moreover, angiogenic processes do not necessarily occur simultaneously with neurogenic processes (Chevillard et al., 2022), which could also explain discrepancies.

Therefore, *in vivo* validation of the presence of AN may be improved by acquisition and cross-correlation of several MR parameters. This is illustrated in Figure 2 with a patchwork of different MR techniques used to determine one or more parameters responding to AN in the sheep hypothalamus during photoperiod. Also, all the knowledge accumulated to date of AN relies on reproducible immunohistochemical findings. Immunohistological-guided MR approaches must therefore be systematically conducted to validate the *in vivo* surrogate markers of AN since the different stages of AN remain invisible to macro-neuroimaging techniques. To illustrate this, rCBVmaps (Figure 2E) and their comparison in the hypothalamus of ewes show a significant increase during the short days of the year (Figure 2F) when AN was detected (Figure 1) (see Just et al., 2021b for methods). BOLD fMRI upon hypercapnic challenge (Figure 2H) during short days (Chevillard et al., 2021) and ^1^H-MRS (Figure 2I) of the hypothalamus demonstrated activation in hypothalamic areas and corresponded well to parallel changes in glutamate, respectively (Figure 2I). We developed a multiple MR experiment protocol in parallel to immunohistochemical experiments for cohorts of ewes of similar age to combine structural, functional, and metabolic MR techniques and histology and obtain *quantitative and reliable surrogate markers* of AN during environmental and physiological cues such as photoperiod. To our knowledge, this is one of the first studies of its kind. We used a cohort of ewes and a routine clinical MR scanner at 3T to determine the cross-sensitivity of VBM, BOLD-fMRI, vascular space occupancy (VASO), dynamic susceptibility contrast MRI, myelin MRI, and ^1^H-MRS to hypothalamic AN. All these MR techniques are widely used in the clinical setting. These examples were extracted from data, which are still under analysis but appear to show some consistent response among all the different MR modalities used with structural, functional, and hemodynamic changes occurring during the period of AN.

## Diffusion-weighted MR methodologies

Diffusion MRI (dMRI) has become an inevitable neuroimaging technique, which has proven to be uniquely sensitive to the presence of various cell types (axons, dendrites, microtubules, and cell bodies) and their density, orientation, and permeability. dMRI provides unprecedented microstructural information on a micrometer scale enabling the investigation of underlying subtle microstructural changes in various disorders and physiological mechanisms. It represents therefore a technique of interest to investigate brain neuroplasticity both in animal models and in the clinical setting (Gatto, 2020). As for the other techniques mentioned above, dMRI has been used to investigate AN mainly in animals models (Vestergaard-Poulsen et al., 2011; Pérès et al., 2018). Vestergaard-Poulsen et al. (2011) demonstrated that dMRI of the hippocampus of rats undergoing chronic stress was able to detect change in water diffusion properties in cell layers of the DG, indicating sensitivity to neurogenesis. Since then, dMRI techniques have improved significantly, enabling increased sensitivity to ever smaller microstructural changes. New dMRI methods such as multidimensional MRI are able to disentangle individual microstructural features (Lundell et al., 2019), which could be powerful for recognizing new developing neurons from mature networks especially if the method is able to identify cell shape and cell size. Interestingly, these methodologies are not restricted to animal models or *ex vivo* investigations with direct possible applications to study the human brain. Finally, diffusion-weighted MR spectroscopy (DWS) uses the diffusion of endogenous metabolites to probe the microstructural features of the intracellular and extracellular spaces. DWS approaches have been shown to be very effective to probe the intra-neural space as well as astrocytic morphology with N-acetyl-aspartate (NAA), glutamate (Glu), and inositol (Ins) both in animal models and human subjects (Palombo et al., 2018; Lundell et al., 2019, 2021).

## Magnetic resonance spectroscopy (MRS)

How brain metabolism influences AN is a question that had gained more and more insights during the past decade. In particular, neuroenergetic mechanisms controlling neural stem cells and progenitor cells and their differentiation, proliferation, and maturation require vast amounts of energy involving various steps and various metabolic mechanisms such as lipogenesis and mitochondrial activity (reviewed by Knobloch and Jessberger, 2017). Notably, It was shown that the balance between glycolysis and oxidative metabolism plays an important role in each stage of AN and may be linked with the development of vasculature. Moreover, an important regulation role for reactive oxygen species (ROS) was also identified during neural stem and progenitor cell (NPC) proliferation and differentiation processes.

Despite their non-invasiveness and translation potential, MRS investigations of AN have been rather limited to date. Studies by Manganas et al. (2007) attracted a lot of interest since the authors claimed to have found a biomarker of NPCs by proton MRS (^1^H-MRS). The methodology proposed by Manganas et al. (2007) went from *in vitro* measurement of the metabolism of NPC cells to their implantation in the hippocampus of rats and *invivo*^1^H-MRS measurement at 9.4 T. In ^1^H-MR spectra, a peak was found at 1.28 ppm, which was significantly elevated compared to control cells or control areas. The analysis of the chemical nature of the 1.28 ppm peak suggested that it could arise from lipid droplets during AN. Finally, measurements were performed on the hippocampus of healthy human subjects at 3 T. Unfortunately, important issues regarding the acquisition and analysis of their data were raised (Friedman, 2008).

Recently, Cherix et al. (2020) demonstrated the existence of a glycolytic switch in the *in ovo* avian retina related to neurogenesis. This study also illustrated how neurogenesis can remodel neuroenergetics and showed the value of MRS in this instance.

From a broader and translational perspective, we performed an ^1^H-MRS at 3 T of the hypothalamus of ewes during photoperiodism to identify potential changes in the neurochemical profile due to neurogenesis. Early results revealed significant changes in NAA, Glu (Figure 2), and Ins during the period of short days corresponding to AN compared to the period of long days (Just et al., 2021b). This preliminary study suggested that *in vivo* MRS is a promising tool to detect the presence of AN or its effects on gyrencephalic brains in the clinical setting. The large size of the voxel of interest prevents a more specific evaluation of neurogenic zones pointing to the need for scanning at higher field strengths and/or editing techniques for increased selectivity of the measured region. Other methodologies such as functional MRS (fMRS) and ^31^P-MRS could be of interest for investigating AN. For example, Koush et al. (2019) showed that j-edited fMRS at 4 T demonstrated good sensitivity and specificity for task-induced lactate modulation as well as for a smaller peak at 1.28 ppm in the motor cortex. These methodological developments could be useful for *in vivo* evaluation of the accumulated changes in lactate seen in other studies on neurogenesis (Prevot et al., 2018; Cherix et al., 2020).

## Considerations on non-neurogenic processes in the adult brain

As mentioned at the beginning of this review, the existence of AN in the human brain remains controversial. In contrast to the well-accepted existence of hippocampal neurogenic niches (DG and SVZ) in the human brain [despite remaining controversies (Kempermann et al., 2018)], the existence of other remodeling plasticity processes is privileged (La Rosa et al., 2020). Over the past decade, despite the tremendous expectations behind the potential of AN for therapeutic strategies targeted at neurodegenerative, neuropsychiatric, and metabolic diseases, investigations have failed to demonstrate significant effects (Bonfanti and Peretto, 2011; La Rosa et al., 2020). These failures may be attributed to the restriction of AN to specific niches and its small quantitative effects as compared to synaptic plasticity, which has been demonstrated to be more consistent and to have important effects on brain damages. Structural and functional plasticity occurs because of modification of the number of synapses, restructuring of dendritic and axonal branches, gliogenesis, and modulation of already existing connections, and pre- and post-synaptic changes, respectively. Both the structural and functional forms of plasticity have been extensively studied with MRI techniques in the adult brain since they occur on a larger scale than AN, and identical mechanisms of synaptic plasticity can be observed in various animal brains as well as in the human brain. However, numerous investigations on synaptic plasticity mechanisms subsist (Martin et al., 2000). All an all, the analysis of the current literature on brain plasticity, whether based on AN or other mechanisms, points toward a need for more thorough, repeatable, and reproducible methodologies and analyses of the human hippocampus starting already from immunohistochemical studies (Kempermann et al., 2018). We also hypothesize that there is a lack of efficient tools to investigate AN *in vivo* and non-invasively in the human brain. Despite the fact that differences in AN development between rodents and humans are acknowledged, findings on rodent neurogenic niches continue to be a point of reference. This is again the case in the mini review of Kempermann et al. (2018). Therefore, other strategies need to be defined since the current neuroimaging spatial and temporal resolutions are limited in the human brain. Immunohistochemical-guided neuroimaging studies conducted on the brain of sheep with naturally occurring AN could prove useful in this instance.

## Conclusion

Adult neurogenesis in the human brain has been described as a key process expected to tackle various neurodegenerative and metabolic diseases but remains controversial. Despite important advances on the topic, sparse developments have been made regarding specific *in vivo* measurements and translational neuroimaging. Here, we argued that an arsenal of MR techniques exists, which, combined with adequate animal models, could pave the way to resolving the controversy on the existence of AN in the human brain.

## Author contributions

NJ wrote the manuscript. P-MC and MM edited, commented, and helped with the figures. All authors contributed to the article and approved the submitted version.

## Funding

This work was funded by the Agence Nationale de la Recherche ANR-16-CE37-0006. NJ received research funding from the Lundbeck Foundation (Experiment grant, grant no. R370-2021-402).

## Conflict of interest

The authors declare that the research was conducted in the absence of any commercial or financial relationships that could be construed as a potential conflict of interest.

## Publisher's note

All claims expressed in this article are solely those of the authors and do not necessarily represent those of their affiliated organizations, or those of the publisher, the editors and the reviewers. Any product that may be evaluated in this article, or claim that may be made by its manufacturer, is not guaranteed or endorsed by the publisher.
